# The static balance ability on soft and hard support surfaces in older adults with mild cognitive impairment

**DOI:** 10.1371/journal.pone.0295569

**Published:** 2023-12-11

**Authors:** Liuxin Qi, Mian Zhou, Min Mao, Jie Yang

**Affiliations:** 1 Graduate School of Education, Shandong Sport University, Jinan, Shandong, China; 2 Medical Department, Weishan County People’s Hospital, Jining, Shandong, China; 3 School of Nursing and Rehabilitation, Cheeloo College of Medicine, Shandong University, Jinan, Shandong, China; 4 College of Sports and Health, Shandong Sport University, Jinan, Shandong, China; Universita Politecnica delle Marche Facolta di Ingegneria, ITALY

## Abstract

**Objective:**

This study aimed to assess the static balance ability of the older adults with mild cognitive impairment (MCI) while standing on soft and hard support surfaces.

**Methods:**

Forty older adults participated in this study (21 in the MCI group and 19 in the control group). Participants were required to perform balance tests under four conditions of standing: standing on a hard support surface with eyes open, standing on a soft support surface with eyes open, standing on a hard support surface with eyes closed, and standing on a soft support surface with eyes closed. Each test was measured in three trials and each trial lasted 30 seconds. Participants were asked to take off their shoes and place their feet in a parallel position with a 20-centimeter distance for bipedal support. The trajectories of the center of pressure (COP) were measured using a Kistler force platform with a frequency of 1000 Hz to assess balance while standing in both groups, with larger COP trajectories indicating poorer static balance in older adults.

**Results:**

With eyes open, the displacement of COP in the anterior-posterior direction(D-ap) (hard support surface: P = 0.003) and the 95% confidence ellipse area(95%AREA-CE) (soft support surface: P = 0.001, hard support surface: P < 0.001) of the COP in the MCI group standing on hard and soft support surfaces were significantly larger than the control group. The 95%AREA-CE (P < 0.001) of the COP in the MCI group on the soft support surface was significantly larger than on the hard support surface. With eyes closed, the root mean square distance(RDIST), root mean square distance-ML(RDIST_ml_), and 95%AREA-CE of the COP were no significant between-group differences when standing on hard support surfaces. However, the RDIST (P = 0.014), RDIST_ml_ (P = 0.014), and 95%AREA-CE (P = 0.001) of the COP in the MCI group on the soft support surfaces were significantly larger than the control group. The 95%AREA-CE (P < 0.001), RDIST (P < 0.001), and RDIST_ml_ (P < 0.001) of the COP in the MCI group on the soft support surface were significantly larger than the hard support surface.

**Conclusion:**

With eyes open, the older adults with MCI showed poorer static balance ability compared to the older adults with normal cognition on soft and hard support surfaces. With eyes closed, the older adults with MCI showed poorer static balance on soft support surfaces, but no differences on hard support surfaces compared with the older adults with normal cognition. With eyes open and closed, the older adults with MCI showed poorer static balance on soft support surfaces as compared to hard support surfaces.

## Introduction

Mild cognitive impairment (MCI), a transitional phase between normal aging and dementia, is considered a risk factor for dementia [1]. The rate of progression to dementia in older adults with MCI is 60%–100% within 5–10 years [2]. Several studies have shown that the older adults with MCI are not only at high risk for future dementia but also for falls [3]. The reason is that their cognitive and executive abilities are decreased, which leads to motor dysfunction, reducing their balance and increasing the risk of falls [4]. The older adults with cognitive impairment have at least twice the risk of falls compared to those with normal cognition [4]. Falls cause severe injuries in older adults with MCI, including soft tissue damage, hip or pelvic fracture, and traumatic brain injuries, which can eventually result in death and bring a huge economic burden to families and society [5, 6]. Therefore, preventing falls in older adults with MCI has become a top priority [7, 8].

Falls in older adults with MCI are caused by many factors, among which poor postural balance is a major risk factor for their falls [9, 10]. An important issue in older adults with cognitive impairment is the higher risk of falls due to impaired static standing balance [11, 12]. A previous study reported that the average absolute maximum velocity (AAMV) of the center of pressure (COP) during standing in older adults with MCI was significantly larger compared to the older adults with normal cognition [13]. Similarly, another study reported that the anterior-posterior sway and area of confidence ellipsis of COP during standing in MCI were significantly larger compared to the older adults with normal cognition [14]. Larger COP sway represented poorer static standing balance in older adults with MCI, which may increase their risk of falls [15]. Consequently, gaining a better understanding of static balance in older adults with MCI may contribute to reducing the risk of falls. The indicator related to the plantar center of pressure trajectories is considered valid for evaluating static postural control. It has been widely used in studies, and the analysis of COP trajectories contributes to understanding static balance control associated with cognitive impairment [16].

Static standing balance is an important motor function that affects the lives of older adults, and its maintenance requires the central nervous system to integrate information from vestibular, visual, and proprioceptive [17]. Studies have shown that the contribution of proprioception and skin sensitivity to the maintenance of standing balance is about 60–70%, while the visual and vestibular systems contribute the rest [17–19]. The above indicated that proprioception played an important role in maintaining static balance [18]. Proprioception may be affected by a variety of factors, among which the support surface is an important factor affecting proprioception, and soft support surfaces can lead to a decrease in balance in older adults [20, 21]. A previous study has reported that older adults had poor static balance with significantly larger sway distances and faster sway velocity of COP while standing on a soft support surface compared to a hard support surface [21]. A recent study also found that the sway displacement and velocity of COP standing on soft support surfaces were significantly larger than on hard-textured and hard support surfaces in older adults [22]. As a population at high risk for falls, early identification of subtle changes in static balance with MCI older adults standing on soft support surfaces may help design targeted interventions to improve their balance. Most previous studies on the balance ability of older adults with MCI focused on hard support surfaces. However, their performance on soft support surfaces remains unclear. Therefore, this study assessed the static balance ability of the older adults with MCI standing on soft and hard support surfaces.

## Materials and methods

### Participants

#### Sample size estimation

G*Power 3.1 software was used to calculate the sample size and the following data were determined: effect size = 1.22 [23], two-tailed significance, statistical power = 0.8, and α value = 0.05. Thus, each group of 12 participants was the required sample size. The determination of effect sizes was based on a previous study that compared the root mean square distance-ML(RDIST_ml_) of the COP in older adults with MCI and the older adults with normal cognition (172.4 ± 50.5 mm vs 110.1 ± 51.8 mm) [23].

#### Recruitment

All participants were recruited between March 10, 2021 and June 20, 2021. A total of 21 older adults with MCI were recruited as the MCI group and 19 older adults with normal cognition were recruited as the control group. The diagnosis of MCI was based on the latest consensus criteria [24], and cognitive impairment was assessed by the Montreal Cognitive Assessment (MoCA). The study was approved by the Exercise Science Ethics Committee of Shandong Sport University (No. 2021006). The study complied with the guidelines of the revised Declaration of Helsinki and all the participants signed a written informed consent statement.

The inclusion criteria for the MCI participants were as follows [25–27]: a recent diagnosis of MCI; aged 65 years and older; the older adults who can walk independently without an assistive device (e.g., cane or walker); MoCA scale score < 26; and corrected visual acuity > 1.0 in both eyes. Meanwhile, in the control group, inclusion criteria were aged 65 years and older; an absence of subjective cognitive complaints, normal objective cognitive testing; no vestibular dysfunction and sensory dysfunction; the older adults who can walk independently without assistive devices; MoCA scale score ≥ 26; and corrected visual acuity > 1.0 in both eyes. Exclusion criteria for both groups were as follows: any neurological disease with motor deficits (e.g., stroke, epilepsy); musculoskeletal system disorders or history of knee or hip replacement surgery affecting normal gait performance; severe depression affecting motor ability; and severe uncorrected visual or auditory impairment.

### Data collection

The Kistler 3D force platform (Switzerland, model 9281CA, 60 cm * 90 cm * 10 cm) was used to collect the displacement data of the COP during standing at a frequency of 1000 Hz [28]. The static balance measurements were conducted in a quiet testing room. Participants were required to perform four balance tests: standing on a hard support surface with eyes open, standing on a soft support surface (5 cm thick foam) with eyes open, standing on a hard support surface with eyes closed, and standing on a soft support surface (5 cm thick foam) with eyes closed [21].

**Fig 1:**
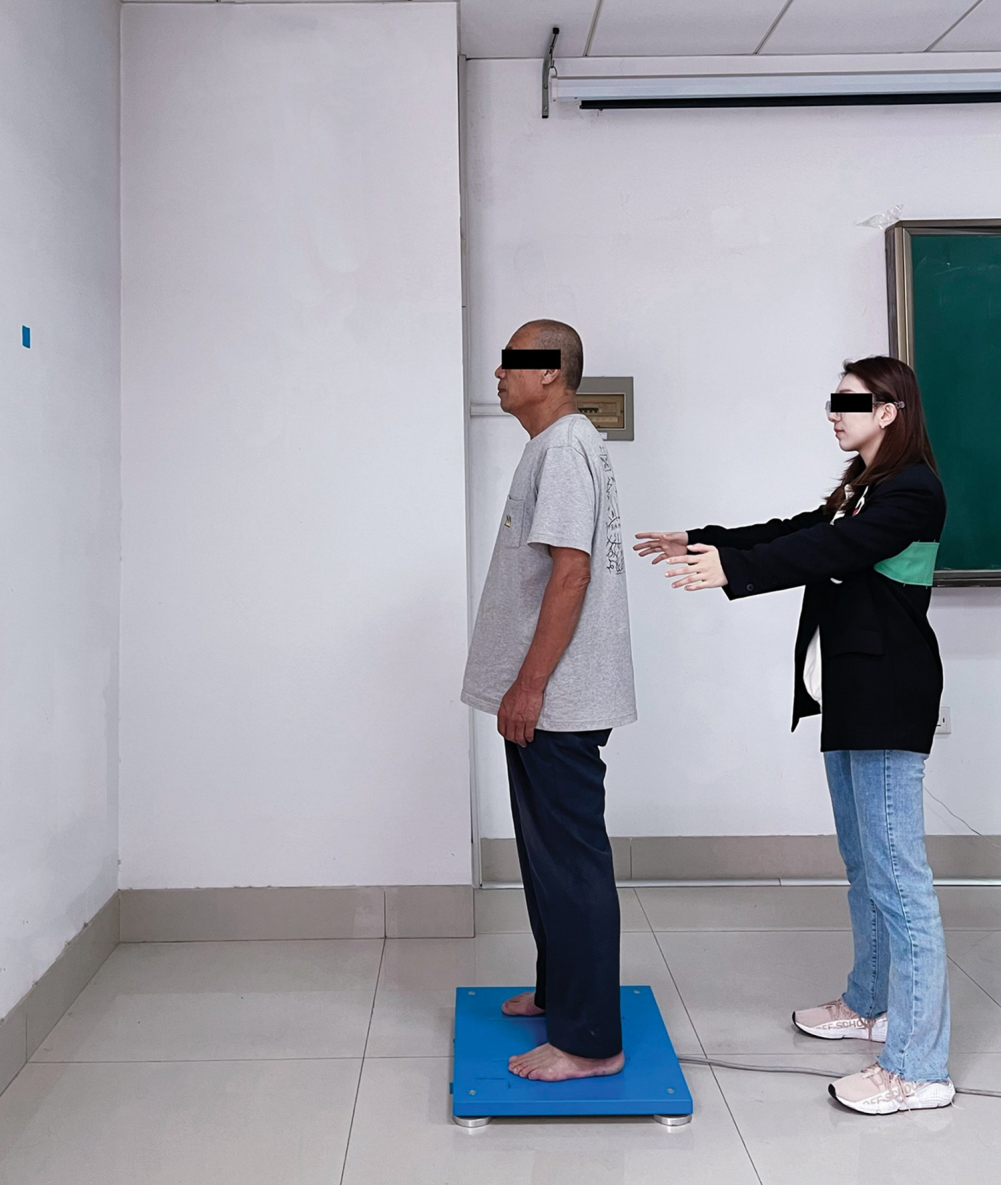
Balance tests on hard support surface.

**Fig 2:**
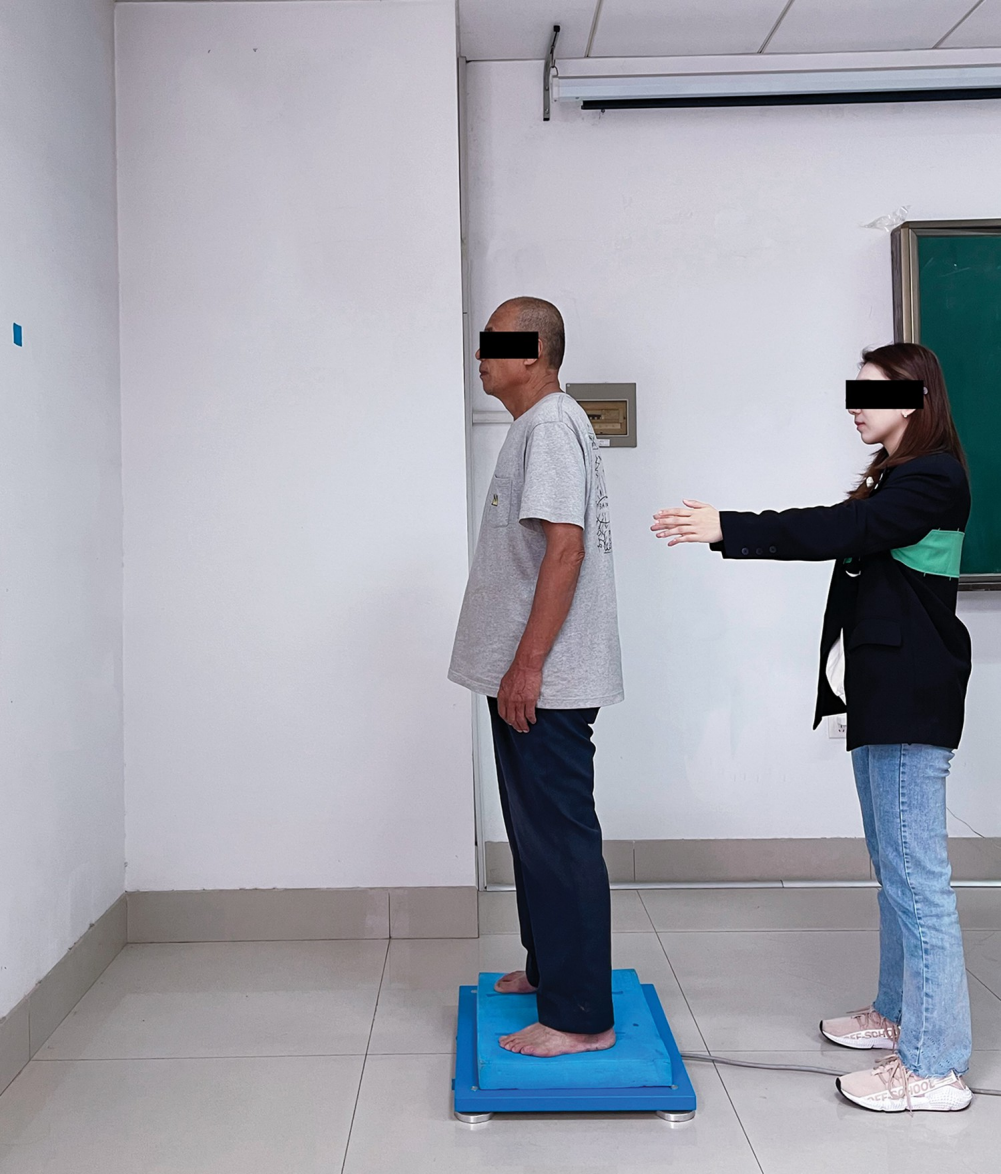
Balance tests on soft support surface.

In addition, each participant was asked to stand barefoot with two feet, which were positioned parallel with a 20 cm distance [26]. They were positioned with arms hanging relaxed to the sides while focusing on a visual reference mark placed in front of them at a 100 cm distance with eyes open [29]. If one leg moved, then the trial failed. Each data was collected for 30 seconds. Participants were given three opportunities to familiarize themselves with the test procedure before the formal measurement. Three successful trials for each balance test were conducted after the procedures were familiarized. The time interval for breaks was 60 seconds between two consecutive tests. The research assistant was always around the participant for protection (Figs 1 and 2).

### Data processing

**Fig 3:**
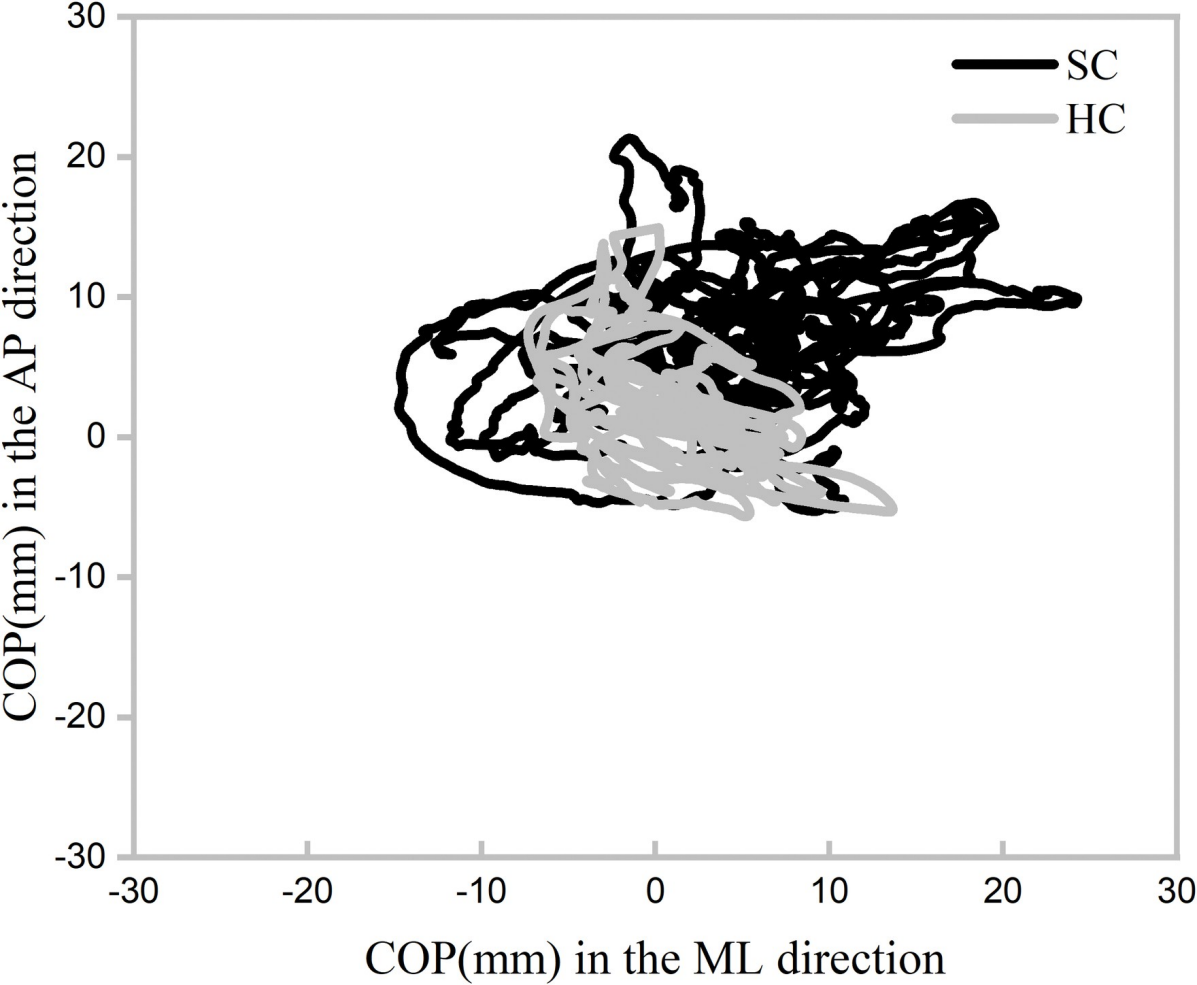
COP trajectories of SC and HC for a representative participant. COP: center of pressure; SC: stood on the soft support surface with eyes closed; HC: stood on the hard support surface with eyes closed; AP: anterior-posterior; ML: medial-lateral.

The data were low-pass filtered with a cutoff frequency of 50 Hz in BIOWARE software [28]. The outcomes were calculated based on the trajectories of the COP to assess the static balance (Fig 3) [30].

D-ml is the maximal displacement of COP in the mediolateral direction.

$$D-ml={ML}_{max}-{ML}_{min}$$

D-ap is the maximal displacement of COP in the AP direction.

$$D-ap={AP}_{max}-{AP}_{min}$$

The root mean square distance (RDIST) from the mean COP is the RMS value of the RD time series. Where the resultant distance (RD) time series is the vector distance from the mean COP to each pair of points in the Apo and MLo time series.

$$RDIST={\left[1/N\sum {{RD}_{\left[n\right]}}^{2}\right]}^{1/2}$$

The root mean square distance-AP (RDIST_ap_) from the mean COP is the standard deviation of the AP time series. Where the mean COP is the position on the force platform defined by the arithmetic means of the APo and MLo time series.

$${RDIST}_{ap}={\left[1/N\sum {{AP}_{\left[n\right]}}^{2}\right]}^{1/2}$$

The root mean square distance-ML (RDIST_ml_) from the mean COP is the standard deviation of the ML time series.

$${RDIST}_{ml}={\left[1/N\sum {{ML}_{\left[n\right]}}^{2}\right]}^{1/2}$$

The 95% confidence ellipse area (95% AREA-CE) is the area of the 95% bivariate confidence ellipse, which is expected to enclose approximately 95% of the points on the COP path.


$$AREA-CE=2\Pi F_{.05[2,n-2]}{\left[S_{AP}^{2}S_{ML}^{2}-S_{APML}^{2}\right]}^{1/2}$$

### Statistical analysis

The SPSS 26.0 statistical software package (IBM SPSS, Armonk, NY, USA) was used for data analysis. All variables were presented as mean ± standard deviation. The independent variables in this study were the group (MCI group and control group) and the type of support surface (hard support surface and soft support surface). Meanwhile, the normality of all outcome variables was tested using the Shapiro-Wilk test, and Two-way ANOVA was used to determine the main effects of groups, support surfaces, and their interaction on the measurements. If any interaction effects were found, the Bonferroni method was conducted for post-hoc comparisons. In addition, partial eta squared (η^2^) was used to represent the effect size of the interaction. The thresholds for Partial eta squared were as follows: 0.01–0.06, small; 0.06–0.14, moderate; and > 0.14, large. Cohen’s d was used to represent the effect size of post-hoc comparisons. The thresholds for Cohen’s d were as follows: < 0.20, trivial; 0.21–0.50, small; 0.51–0.80, medium; and > 0.81, large [31]. The significance level was set at 0.05 and the extreme significance level was set at less than 0.001.

## Results

### Baseline characteristics of the participants

A total of 40 participants were screened for eligibility, 21 in the MCI group and 19 in the control group, respectively. All participants conducted the final balance test. The Shapiro-Wilk test confirms that the variables are normally distributed. The basic characteristics of participants were compared using the Independent-Samples t-test, which showed a significant difference between the MoCA scores of the MCI group and control group as expected (p < 0.05). There were no significant differences found in age, height, weight, and education years between the two groups. The characteristics of the participants are shown in Table 1.

**Table 1:** Baseline characteristics of participants.

	Controls(n = 19)	MCI(n = 21)	P value
**Age(years)**	68.42±4.80	71.00±3.86	0.068
**Height(cm)**	163.53±6.63	159.71±7.29	0.055
**Weight(kg)**	64.46±8.07	63.61±10.04	0.771
**Education years(years)**	6.05±2.93	4.20±3.22	0.053
**MoCA(scores)**	26.47±0.90	18.33±2.97	<0.001

### The static balance ability with eyes open

As shown in Table 2, Two-way ANOVA analysis showed significant interaction effects in D-ap (P = 0.028, η^2^_p_ = 0.062), 95%AREA-CE (P = 0.007, η^2^_p_ = 0.093). Significant group effects and support surface effects were found in our study. Post-hoc analysis showed that the D-ap (P = 0.003, Cohen’s d = 1.04), 95%AREA-CE (P < 0.001, Cohen’s d = 1.35) in the MCI group on the hard support surface were significantly larger than the control group. The 95%AREA-CE (P = 0.001, Cohen’s d = 1.17) in the MCI group on the soft support surface was significantly larger than the control group. Meanwhile, the 95%AREA-CE (P < 0.001, Cohen’s d = 1.53) in the MCI group on the soft support surface was significantly larger than on the hard support surface.

**Table 2:** Comparison of static balance ability with eyes open between MCI group and control group.

	MCI group		Control group		group		support surface		group × support surface	
	hard support surface	soft support surface	hard support surface	soft support surface	P value	η^2^_p_	P value	η^2^_p_	P value	η^2^_p_
**D-ml (mm)**	**12.47±5.58**	**24.67±4.11**	**8.30±2.92**	**18.43±3.75**	**<0.001**	**0.283**	**<0.001**	**0.645**	**0.278**	**0.015**
**D-ap (mm)**	**10.51±8.45** ^**a**^	**12.71±2.41**	**4.19±1.62**	**11.59±5.02** ^**b**^	**0.002**	**0.119**	**<0.001**	**0.184**	**0.028**	**0.062**
**RDIST (mm)**	**1.61±0.41**	**3.50±1.30**	**1.24±0.30**	**2.65±0.84**	**0.001**	**0.128**	**<0.001**	**0.515**	**0.196**	**0.022**
**RDIST**_**ml**_ **(mm)**	**1.33±0.31**	**3.02±1.12**	**1.09±0.29**	**2.22±0.70**	**0.001**	**0.129**	**<0.001**	**0.515**	**0.080**	**0.040**
**RDIST**_**ap**_ **(mm)**	**0.96±0.43**	**1.68±0.61**	**0.50±0.10**	**1.41±0.54**	**0.001**	**0.137**	**<0.001**	**0.443**	**0.368**	**0.011**
**95%AREA-CE (mm** ^**2**^ **)**	**58.17±15.25** ^**a**^	**211.37±106.56** ^**ab**^	**37.54±15.40**	**116.19±43.52** ^**b**^	**<0.001**	**0.198**	**<0.001**	**0.498**	**0.007**	**0.093**

Abbreviations: D-ml, the maximal displacement of the COP in the medial-lateral direction; D-ap, the maximal displacement of the COP in the anterior-posterior direction; RDIST, the root mean square distance of the COP; RDIST_ml_, the root mean square distance of the COP in the medial-lateral direction; RDIST_ap_, the root mean square distance of the COP in the anterior-posterior direction; 95%AREA-CE, the 95% confidence ellipse area of the COP.

^a^ significant between-group differences on the same support surface.

^b^ significant within-group differences on the different support surfaces.

### The static balance ability with eyes closed

As shown in Table 3, Two-way ANOVA analysis showed significant interaction effects in 95%AREA-CE (P = 0.001, η^2^_p_ = 0.13), RDIST (P = 0.047, η^2^_p_ = 0.051), and RDIST_ml_ (P = 0.041, η^2^_p_ = 0.054). Significant group effects and support surface effects were found in our study. Post-hoc analysis showed that there were no significant between-group differences found in the 95%AREA-CE, RDIST, and RDIST_ml_ in the MCI group on the hard support surface compared to the control group. The 95%AREA-CE (P = 0.001, Cohen’s d = 1.14), RDIST (P = 0.014, Cohen’s d = 0.79), and RDIST_ml_ (P = 0.014, Cohen’s d = 0.80) in MCI group on the soft support surface significantly larger than the control group. Meanwhile, the 95%AREA-CE (P < 0.001, Cohen’s d = 3.46), RDIST (P < 0.001, Cohen’s d = 2.35), and RDIST_ml_ (P < 0.001, Cohen’s d = 2.50) in MCI group on the soft support surface was significantly larger than the hard support surface.

**Table 3:** Comparison of static balance ability with eyes closed between MCI group and control group.

	MCI group		Control group		group		support surface		group × support surface	
	hard support surface	soft support surface	hard support surface	soft support surface	P value	η^2^_p_	P value	η^2^_p_	P value	η^2^_p_
**D-ml (mm)**	**13.69±5.21**	**44.58±14.07**	**11.70±4.68**	**33.96±11.30**	**0.005**	**0.099**	**<0.001**	**0.662**	**0.051**	**0.049**
**D-ap (mm)**	**7.73±5.20**	**20.62±8.25**	**4.64±1.74**	**17.09±7.64**	**0.021**	**0.068**	**<0.001**	**0.516**	**0.876**	**0.000**
**RDIST (mm)**	**1.91±0.63**	**6.98±2.40** ^a^ ^b^	**1.60±0.46**	**5.32±1.53** ^b^	**0.004**	**0.102**	**<0.001**	**0.694**	**0.047**	**0.051**
**RDIST**_**ml**_ **(mm)**	**1.70±0.57**	**6.14±1.99** ^a^ ^b^	**1.48±0.43**	**4.73±1.40** ^b^	**0.006**	**0.096**	**<0.001**	**0.704**	**0.041**	**0.054**
**RDIST**_**ap**_ **(mm)**	**0.82±0.41**	**2.92±1.78**	**0.56±0.16**	**2.34±1.02**	**0.084**	**0.039**	**<0.001**	**0.467**	**0.508**	**0.006**
**95%AREA-CE (mm** ^**2**^ **)**	**90.67±50.35**	**949.48±268.87** ^**ab**^	**68.55±36.87**	**611.90±315.88** ^**b**^	**<0.001**	**0.163**	**<0.001**	**0.747**	**0.001**	**0.130**

Abbreviations: D-ml, the maximal displacement of the COP in the medial-lateral direction; D-ap, the maximal displacement of the COP in the anterior-posterior direction; RDIST, the root mean square distance of the COP; RDIST_ml_, the root mean square distance of the COP in the medial-lateral direction; RDIST_ap_, the root mean square distance of the COP in the anterior-posterior direction; 95%AREA-CE, the 95% confidence ellipse area of the COP.

^a^ significant between-group differences on the same support surface.

^b^ significant within-group differences on the different support surfaces.

## Discussion

The results showed that with eyes open, the variables assessing the static balance ability in the MCI group standing on the hard support surface, including D-ap and 95%AREA-CE of the COP, were significantly larger than the control group, which is consistent with previous studies [26]. These results might indicate that the static balance ability of the older adults with MCI standing on hard support surface was poorer compared to the older adults with normal cognition in the eyes open condition, which may be related to the significant changes in the white matter and the functional connectivity of Cortical Vestibular Network (CVN) [26, 32]. A recent magnetic resonance imaging study showed that the CVN, a key brain region that integrates visual, auditory, and vestibular sensory information in older adults with MCI [33], was significantly correlated with their poorer balance function [32]. Its alterations might disrupt structural brain connectivity and interfere with neural pathways that control balance, resulting in the inability of the Central Nervous System (CNS) to effectively access or integrate sensory information, which may be the reason for the poorer balance in older adults with MCI [32]. In contrast, our results showed that no significant difference between the two groups on the hard support surface with eyes closed which might indicate that the amount of compensation is similar in the two groups with eyes closed [26]. MCI older adults may have a relatively complete compensation system on the hard support surface with eyes closed, which may be an important finding for guiding their balance training [26].

However, it is noteworthy that the RDIST, RDIST_ml_, and 95%AREA-CE in the MCI group on the soft support surfaces were significantly larger compared to the control group. It might indicate that the static balance on soft support surfaces was poorer than that of the older adults with normal cognition in the eyes closed condition. Previous study has found that maintaining balance relies on the accurate visual, vestibular, and proprioceptive perception of the external environment and information input, and that the relative weight of these information inputs depends on the specific task and environmental context [34]. In conditions without visual and proprioceptive information inputs, the weight of the vestibular increases, and the body balance relies mainly on the vestibular for regulation [35]. The use of soft foam cushions in this study might increase the interference with their proprioception when participants close their eyes to block the input of visual information. The results indicated that the static balance of the MCI group was poorer than that of the older adults with normal cognitions, which may be related to the impaired vestibular system of the older adults with MCI [36]. It is well known that the vestibular system is a complex system consisting of the peripheral nervous system (vestibular organs and vestibular nerves) and the central vestibular system, which plays an important role in maintaining balance [37]. Previous studies have shown that a potential positive correlation exists between vestibular function and cognition, with the more severe the cognitive impairment, the more severe the impairment of vestibular function [36, 38]. And a recent study also has shown that cognitive impairment is primarily associated with impaired otolithic function (vestibular organ), as evidenced by a significant delay in p13 latency on the vestibular-evoked myogenic potentials (VEMP) test in patients with severe cognitive impairment [36]. Thus, impairment of the vestibular organs could lead to an inability to correctly perceive head position information in older adults with MCI, affecting their balance [36].

The results showed that with eyes open and closed, the variables assessing the static balance ability in the MCI group standing on soft support surfaces, including the RDIST, RDIST_ml_, and 95%AREA-CE of the COP, were significantly larger as compared to hard support surfaces. The results demonstrated that the static balance ability of the older adults with MCI standing on soft support surfaces was poorer as compared to hard support surfaces, which is consistent with previous studies [22]. Palazzo’s study showed that the older adults had poor static balance with significantly larger sway distances and faster sway velocity of COP standing on a soft surface as compared to the hard support surface [22]. Proprioception is an important factor in the control of balance in older adults [39]. Standing on soft surfaces might impair the inputs from the joint receptors and skin mechanoreceptors of the foot, and fewer or incorrect signals are transmitted to the brain, resulting in slower or abnormal processing of information received by the central nervous system, which weakens the human body’s ability to respond to external disturbances [22, 35]. In addition, muscle strength plays an important role in controlling body balance [8]. It has been reported that lower muscle strength is associated with a higher risk of developing MCI [40, 41]. Compared to the older adults with normal cognition, the older adults with MCI may have musculoskeletal disorders such as muscle atrophy and hypomuscular strength, resulting in decreased control of proximal and distal muscles of their lower extremities and decreased proprioceptive function, which might affect the control of their balance function [26, 40]. These factors might lead to poor balance ability in the elderly with MCI on soft support surface.

The study had two limitations. Firstly, only 40 participants completed the present study, so the findings should be interpreted with caution. Further study with large sample sizes could conducted. Secondly, this study only collected COP data and didn’t explore the correlations between mild cognitive impairment (MCI) and other factors such as neural network activity and muscle strength in the brain, which limits our understanding of the mechanisms of integrated neuromuscular control of static balance in patients with MCI. Future studies could incorporate additional measurements such as electromyography (EMG) and electroencephalography (EEG) data to provide a more comprehensive analysis of the neuromuscular control mechanisms involved in MCI-related static balance deficits, which would allow us to better understand the complex interactions between cognitive function, neural activity, and muscular control to maintain balance in patients with mild cognitive impairment.

## Conclusions

Our study indicated that the older adults with MCI showed poorer static balance ability compared to the older adults with normal cognition on soft and hard support surfaces with eyes open. Static balance was similar between the older adults with MCI and the older adults with normal cognition in the absence of visual information compensation on the hard support surface, while the older adults with MCI had a poorer static balance on the soft support surface. Meanwhile, the static balance ability of the older adults with MCI standing on soft support surfaces was poorer as compared to hard support surfaces with eyes open and closed.
